# Three-dimensional computational model simulating the fracture healing process with both biphasic poroelastic finite element analysis and fuzzy logic control

**DOI:** 10.1038/s41598-018-25229-7

**Published:** 2018-04-30

**Authors:** Monan Wang, Ning Yang

**Affiliations:** Mechanical & Power Engineering College, Harbin University of Science and Technology, Harbin, Heilongjiang 150080 China

## Abstract

A dynamic model regulated by both biphasic poroelastic finite element analysis and fuzzy logic control was established. Fuzzy logic control was an easy and comprehensive way to simulate the tissue differentiation process, and it is convenient for researchers and medical experts to communicate with one another to change the fuzzy logic rules and improve the simulation of the tissue differentiation process. In this study, a three-dimensional fracture healing model with two different interfragmentary movements (case A: 0.25 mm and case B: 1.25 mm) was analysed with the new set-up computational model. As the healing process proceeded, both simulated interfragmentary movements predicted a decrease and the time that the decrease started for case B was later than that for case A. Compared with experimental results, both cases corresponded with experimental data well. The newly established dynamic model can simulate the healing process under different mechanical environments and has the potential to extend to the multiscale healing model, which is essential for reducing the animal experiments and helping to characterise the complex dynamic interaction between tissue differentiations within the callus region.

## Introduction

Bones are vital organs in humans. Bones provide structural support, as well as physical protection^1^. However, bone fractures often occur in our daily life, especially for the elderly people. Unfortunately, despite the special capacity of self-regeneration and scarless formation of bone, there are still instances in which delayed healing or non-unions can happen, such as pathological fractures or fractures with large defects. Traditional treatment strategies for fractures are primarily dependent on the experience of the orthopedist. The disadvantage of this is that among the many treatment strategies, we cannot predict the treatment effects in advance and choose the best strategy for patients. With the help of the simulation of bone fracture healing, different treatment strategies can be predicted, and the optimal strategy can be chosen, which then can reduce the healing time and lighten the economic burden and pain for the patients.

**Figure 1:**
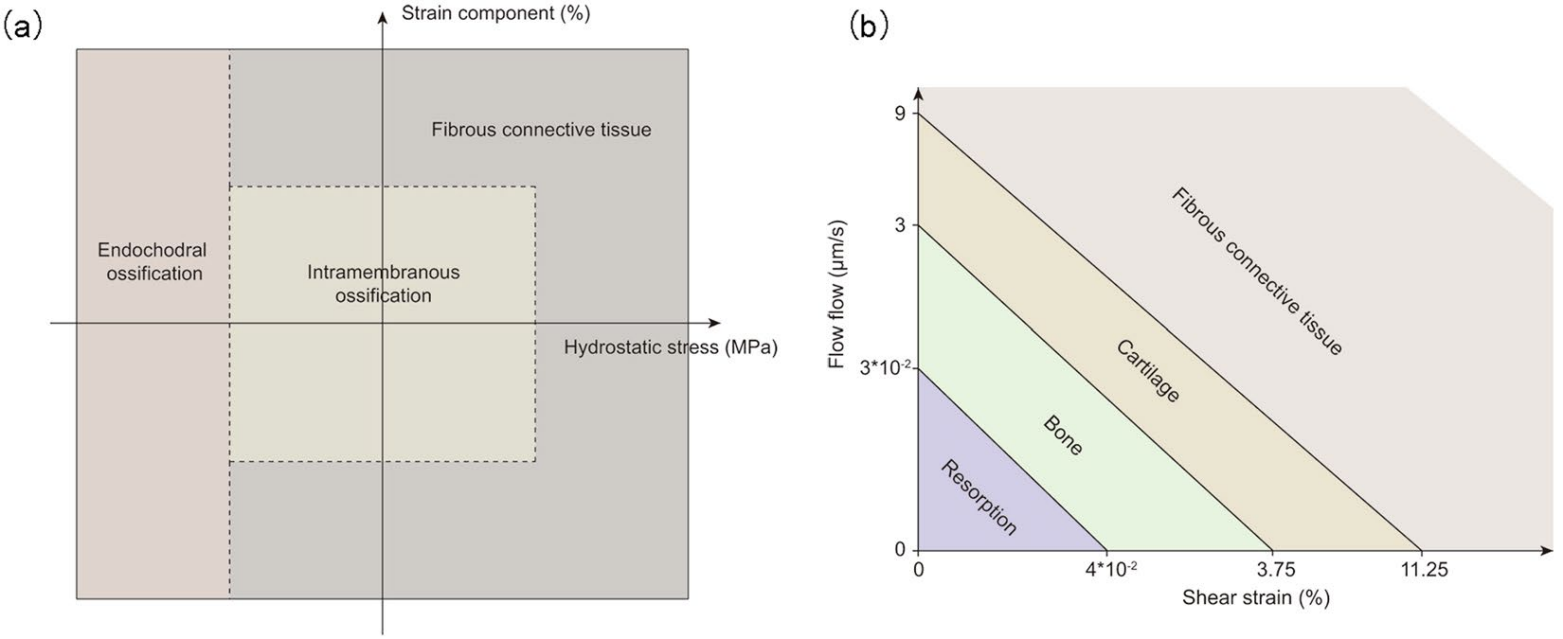
Tissue differentiation pathways regulated by mechanical stimuli. (**a**) Tissue differentiation pathways regulated by hydrostatic stress and strain in single finite element analysis^10^. (**b**) Tissue differentiation pathways regulated by shear strain and fluid flow in biphasic poroelastic finite element analysis^20^.

Over the last several decades, a number of computational simulations of bone fracture healing have emerged. To sum up, there are three types of fracture healing models: mechanoregulatory healing models, bioregulatory healing models and coupled mechanobioregulatory healing models^2^. Mechanoregulatory models were first proposed, in which model mechanical stimuli was the main regulator for the tissue differentiation in the callus region and finite element analysis was used to calculate the mechanical stimuli. Single finite element models and biphasic finite element models were the two primary types used to calculate the mechanical stimuli. In single finite element models^3–9^, tissue differentiation pathways were regulated by mechanical stimuli (Fig. 1a) based on the work of Claes and Heigele^10^ and fuzzy logic control was used to simulate the process of tissue differentiation within the callus region, which is an easy way to establish the healing process as linguistic principles. In biphasic finite element models^11–19^, tissue differentiation pathways regulated by mechanical stimuli (Fig. 1b) based on the work of Huiskes *et al*.^20^ and partial differential equations were used to simulate the healing process by modelling cell activities (proliferation, differentiation). In addition, in the work of Isaksson *et al*.^19^, cell migration was modeled as a diffusion process.

There are still several bioregulatory healing models and coupled mechanobioregulatory healing models. For bioregulatory and coupled mechanobioregulatory models, the healing process was simulated with the activities (migration, proliferation, differentiation and death) of different cells participating in the process within the callus region. These cell activities were regulated by the corresponding growth factors^21–24^ and mechanical stimuli^25,26^. Partial differential equations were used to simulate different cell populations during the healing process, and finite element analysis was used to calculate the local mechanical stimuli. Carlier and her colleagues made a step further in this field, they simulated the healing process with a multiscale modelling method^27,28^ from the intracellular level to the tissue level, which was a more mechanistic modelling method. Although bioregulatory and coupled mechanobioregulatory healing models simulated fracture healing in a more mechanistic way, there are still challenges in this field, such as the transduction of mechanical stimuli from the intracellular level to the tissue level and the influence of mechanical stimuli on growth factors.

The aim of this study was to set up a three-dimensional dynamic healing model regulated by both biphasic finite element analysis and fuzzy logic control. Unlike previous models, our model has the following advantages:

1. In our model, we used a three-dimensional transvers fracture model. Compared with two-dimensional models, we obtained the three-dimensional space distribution of biophysical stimulus, bone concentration, cartilage concentration and blood perfusion with time within the three-dimensional callus region.
2. To verified the predicted results, we made a comparison of our model with experimental data measured by Claes *et al*.^29^ and computational simulation conducted by Wang *et al*.^9^, which simulated the fracture healing process with linear elastic analysis and fuzzy logic. Through comparison, we can conclude that under the condition of large interfragmentary movement (IFM), the predicted IFM curve of our model is closer to the average experimental data. In addition, under the same simulation conditions, the computational time of our model is faster than that of Wang’s model.
3. Through combing biphasic finite element model and fuzzy logic control, the model has its advantages in model extension and model analysis speed.

## Results

In our work, we analyzed fracture healing with two different IFMs with our computational models, which corresponded to two experimental fracture healing cases in sheep^29^. Case A was defined as the more stable situation, whose IFM was 0.25 mm and gap size was 2.1 mm when in the unloaded situation. Case B was defined as the less stable situation, whose IFM was 1.25 mm and gap size was 3.1 mm when in the unloaded situation. Then through the simulation, we compared the calculated IFM with corresponding weekly measured axial movements from previous experiment work^29^. In addition, to make the tissue differentiation taking place with the callus region, the max diameter of the callus was 16 mm, which was according to the work of Claes and Heigele^10^.

**Figure 2:**
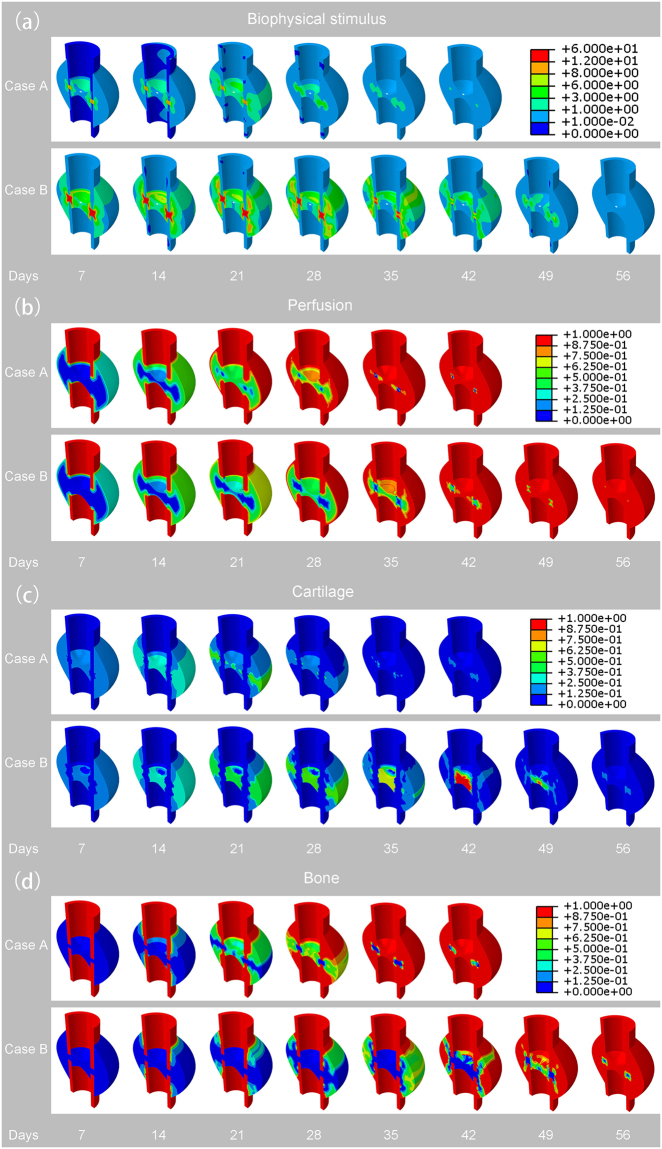
(**a**) Predicted biophysical stimulus for both cases A and B at an interval of 7 days. (**b**) Predicted perfusion for both cases A and B at an interval of 7 days. (**c**) Predicted cartilage concentration for both cases A and B at an interval of 7 days. (**d**) Predicted bone concentration for both cases A and B at an interval of 7 days.

**Figure 3:**
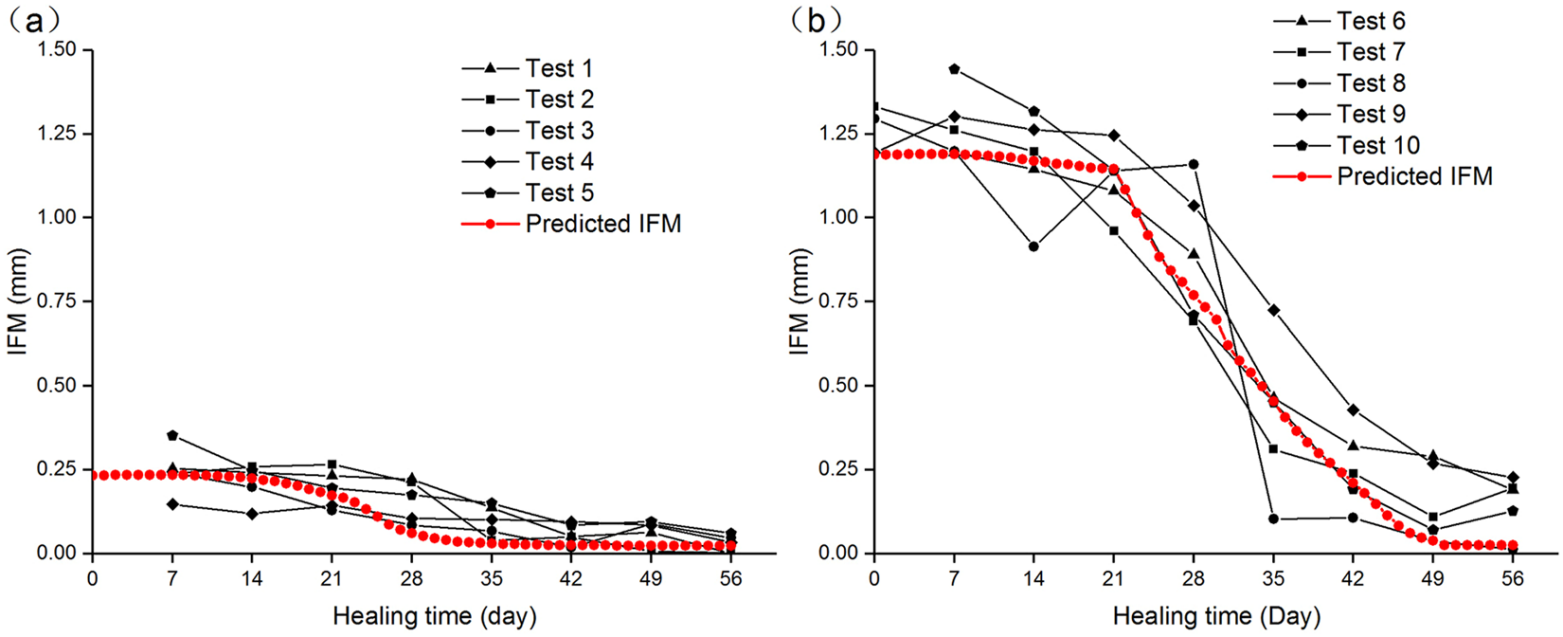
(**a**) Predicted IFM over time of case A and weekly measured IFM from experiments conducted by Claes *et al*.^29^. (**b**) Predicted IFM over time of case B and weekly measured IFM from experiments conducted by Claes *et al*.^29^.

From the simulation results, local biophysical stimulus, blood perfusion, tissue concentrations (cartilage concentration and bone concentration) and IFM were predicted over both time and space for both cases A and B at intervals of 7 days (Figs 2 and 3). The predicted IFM (Fig. 2) produced good prediction results compared with the animal experiments conducted by Claes *et al*.^29^.

The simulation results predicted a larger biophysical stimulus in case B than case A due to the lager IFM that case B subjected. The highest stimulus was predicted in the interfragmentary gap at the initial healing phases for both cases, respectively and the greater values were reached for case B than for case A. As the healing process proceeded, the biophysical stimulus decreased gradually to the physiological values that can be observed from Fig. 2a. The day that case A decreased to the physiological value was at day 42. And the day that case B decreased to the physiological value was at day 56. Therefore, it can be concluded that the increase of IFM delays the time of biophysical stimulus decreasing to physiological values.

There was a slower process of angiogenesis for case B (Fig. 2b). At the initial phases (before day 14), we could see that the angiogenesis was starting at the cortex away from the gap and peripheral side for both cases A and B. Next, the angiogenesis grew towards the gap centre and gradually filled the whole callus region. However, because the biophysical stimulus that case B subjected was over the range of fuzzy logic value “destructive”, the time of angiogenesis for case B was slower for case A. From Fig. 2b, we can see that the time of angiogenesis for case A was at day 42 and for case B, it was ended at day 56.

More cartilage tissue was predicted in case A than in case B (Fig. 2c). In the early phases, cartilage formed in both periosteal and endosteal callus for both cases A and B, where there were higher biophysical stimuli and fewer blood perfusions, which was called the chondrogenesis process. As the healing process proceeded, because the process of endochondral ossification process took place, the formed cartilage differentiated into bone tissue. Therefore, cartilage tisssue began to decrease gradually to disappear. From the Fig. 2c, we can observe that the duration time of cartilage in case B (day 56) was longer than that in case A (day 42), which is because the lager biophysical stimulus experienced by case B was not suitable for the formation of bone.

The new bone formed later in case B than in case A (Fig. 2d). For both cases, intramembranous ossification took place at first near the cortex far away from the gap where there were low biophysical stimuli and proper blood perfusion. Then when the biophysical stimuli and perfusion were proper, the bone formation grew towards the central part of the callus region and the ossification type changed from intramembranous to endochondral ossification. From the Fig. 2d, we could see that the time of bone formation in case B (day 56) was later than in case A (day 42) due to the large fracture gap that resulted in the larger biophysical stimulus and insufficient blood perfusion in case B.

The predicted IFM from the computational simulation is shown in Fig. 3. The IFM curves predicted a decrease for both cases A and B. In the earlier days, there were large IFM values and almost no decrease regarding the curves because during this period, there is little bone formation within the callus region and the formation of cartilage could not provide a stable environment for fracture callus. As the healing proceeded, both curves predicted a rapid decrease due to bone formation. However, because of the higher mechanical stimuli suffered for case B, the time of IFM reaching the minimum value (day 50) was later than that for case A (day 55). Compared with the animal experiments conducted by Claes *et al*.^29^, both IFMs had a good correspondence with the measured experimental results.

**Figure 4:**
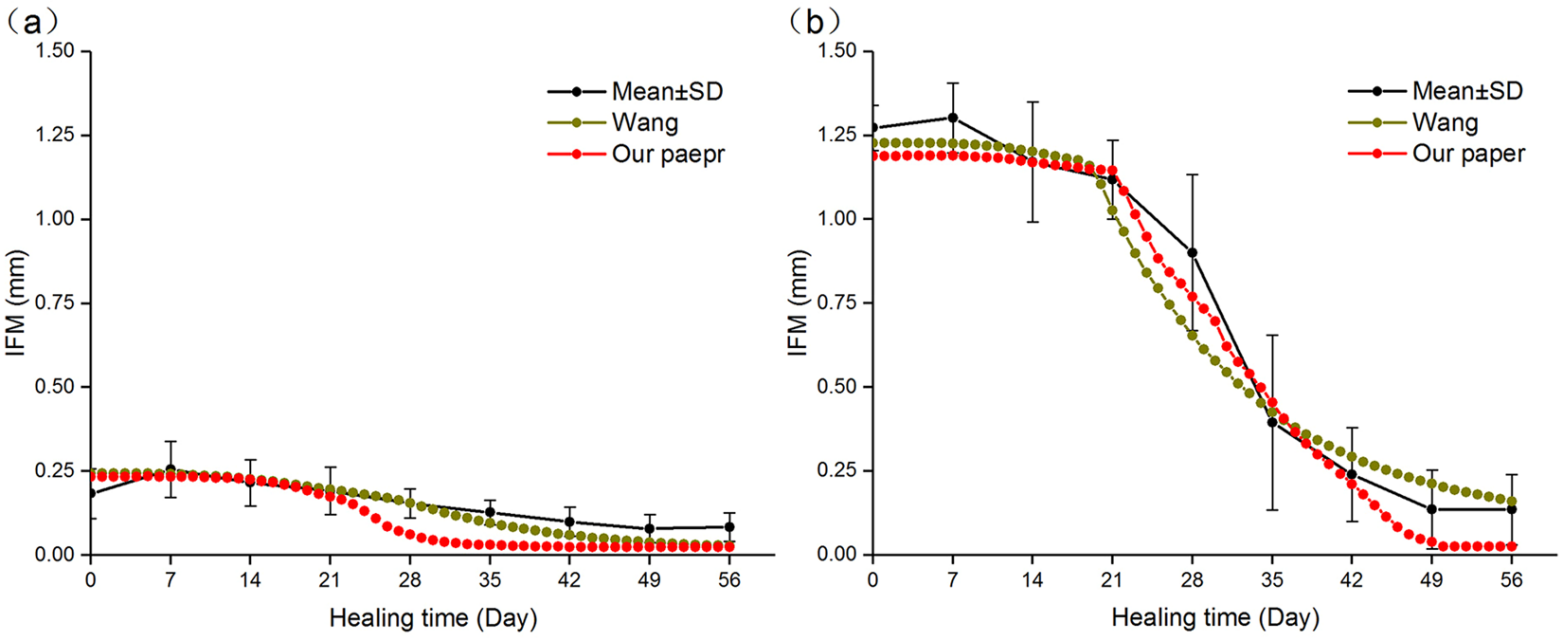
Comparison of IFM between average values of experimental data, Wang’s simulated results and our simulated results.

**Figure 5:**
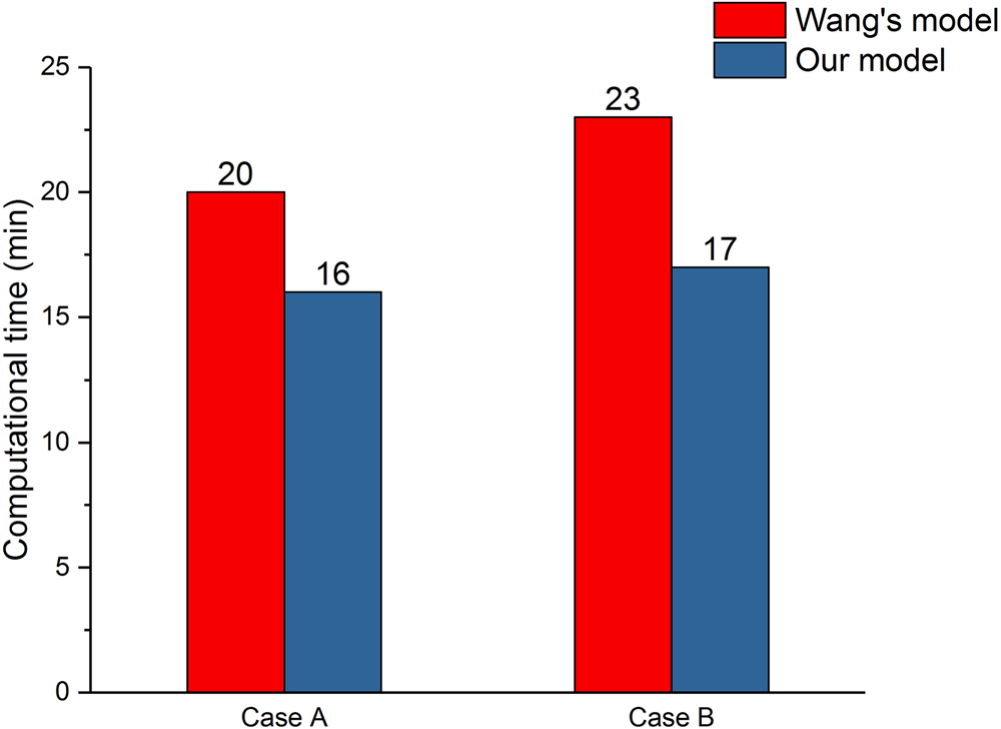
Comparison of computational time between Wang’s model^9^ and our model for both cases A and B.

We compared our model with experimental data and the work of Wang *et al*.^9^. From Fig. 4, we can concluded that the predicted IFM curves of both our model and Wang’s model corresponds trend of experimental data and the predicted data is within the error line basically. In Case A (Fig. 4a), the curves decrease at day 21 for both our model and Wang’s model. However, our model curve descends faster than Wang’s model curve. In Case B (Fig. 4b), both our model and Wang’s model correspond the experimental data well and compared with Wang’s model, the predicted data of our model is closer to the average value of experimental data than that of Wang’s model. In addition, we can conclude from Fig. 5 that under the same simulation conditions, the computational time of our model is faster than that of Wang’s model.

In order to prove that our model is faster than that of Wang’s model, the computational time of both models was simulated under different simulation conditions (different element types and different element numbers) at the same computer configuration (core: 28, threads: 56 RAM: 112 G). For every simulation condition, we simulated three times and recorded their computational time respectively and their mean values. From Tables 1 and 2, we can see that under the same element type and element numbers, the computational time of our model is faster than that of Wang’s model. Therefore, we can conclude that our model has an advantage in computational time that Wang’s model.

**Table 1:** Comparison of computational time between our model and Wang’s model^9^ under Case A.

Element type	Element numbers	Computational time of our model (min)				Computational time of Wang’s model (min)			
		1	2	3	mean	1	2	3	mean
Tetrahedral elements numbers	11642	15	18	18	17	22	24	22	22.7
	17952	20	21	20	20.3	25	25	24	24.7
	32081	25	25	27	25.7	32	30	30	30.7
	43053	28	29	28	28.3	35	35	36	35.3
Tetrahedral elements numbers	10179	16	13	15	14.7	19	19	18	18.7
	13688	17	16	15	16	19	21	20	20
	16590	19	20	18	19	25	24	26	25
	17952	21	19	23	21	28	28	26	27.3

**Table 2:** Comparison of computational time between our model and Wang’s^9^ model under Case B.

Element type	Element numbers	Computational time of our model (min)				Computational time of Wang’s model (min)			
		1	2	3	mean	1	2	3	mean
Tetrahedral elements numbers	12056	15	17	16	16	23	23	23	23
	16840	19	19	17	18.3	26	28	26	26.7
	34988	28	26	28	27.3	32	32	31	31.7
	42938	30	32	31	31	38	36	38	37.3
Tetrahedral elements numbers	11853	13	15	13	13.7	19	19	17	18.3
	15225	16	18	17	17	22	25	22	23
	16830	19	17	19	18.3	25	25	26	25.3
	19107	22	21	21	21.3	27	26	28	27

## Discussion

In this study, we established a dynamic three-dimensional fracture healing model with biphasic poroelastic finite element analysis and fuzzy logic control. With this new model, we can predict the local biophysical stimulus and different tissue distribution within the callus region. The IFM can also be predicted per day. When compared with experimental observations, the model had a good prediction outcome. The newly established model has the following strengths, which are shown as follows:

1. In our work, a three-dimensional computational model was used to simulate the fracture healing. With the three-dimensional callus model, we can obtained an intact information of spatial and temporal distribution for biophysical stimulus, cartilage concentration, bone concentration and blood perfusion.
2. When compared with Wang’s model^9^, we can concluded that in Case B (Fig. 4b), our model curve is closer to the experimental curve between days 21 and 49, which is the most obvious time for fracture healing. This may be due to the different tissue differentiation pathway we chose (our model: Fig. 1a, Wang’s model: Fig. 1b). Therefore, under the condition of big IFM, our model is more accurate than Wang’s model. In addition, because of the different mechanical stimulus we chose, fuzzy logic rules that was used to simulate the tissue differentiation process decreased from 21 to 14. The reduction of fuzzy logic rules reduces the computational analysis time. Therefore, under the same simulation conditions, the computational time of our model is faster than that of Wang’s model.
3. In our work, we used biphasic finite element model to obtain shear strain and fluid flow. Then according Eq. 1, the biophysical stimulus was calculated. The reason for using biphasic finite element model was that in the work of Isaksson *et al*.^16^, through the comparison with three mechanoregulatory models^10,11,30^, there researchers concluded that the model regulated by deviatoric strain and fluid velocity was the most precious, and only this model could predict the healing process under torsional rotation conditions. In these researchers’ further study^17^, they demonstrated that the model regulated by deviatoric strain and fluid flow had a good outcome compared with the experiment results. In addition, fluid shear strain was supposed to be the mechanical signals that is sensed by bone cells and regulate the cell activities, which is the called the mechanotransduction process^31^. Therefore, with biphasic finite element model, we can model the mechanotransduction process and investigate how mechanical signals from tissue level sensed by related cells and regulate the cell response. Therefore, from this aspect, these advantages make the model have a good extension.

Fuzzy logic and coupled partial differential equations are two ways to simulate tissue differentiation. Since the numerical solution of partial differential equations is based on the grid, the increase of the number of meshes can increase the time of solving partial differential equations. In addition, the number of equations used to describe the process of fracture healing will increase with further study of the simulation of fracture healing process, for example the mechanotransduction modeling. This will also increase the analysis time. Therefore, the fuzzy logic method is adopted on the premise of ensuring the conditions of the three-dimensional geometric model and the appropriate model analysis time. In addition, the fuzzy logic rules can easily integrate the biological acknowledgement into rules that are convenient for those who are not familiar with differential equations. With fuzzy logic rules, researchers can easily communicate with medical or biological experts and change the rules conveniently making them easy to maintain and change.

Although the dynamic model has its strengths, there are still several limitations that need to be improved in the future:

1. One limitation of this study was the simulation of angiogenesis. Angiogenesis is a key process during the healing process. This process is a prerequisite for bone formation^32^. In this study, we assumed angiogenesis as a continuous function. When the simulation of the model finished, perfusion was filled with the whole callus region. However, this is not in accordance with the discrete nature of angiogenesis. Therefore, how to integrate the discrete nature of angiogenesis was one of the future works in our study. Some previous models^24,27,28^ had tried to add this nature in their studies. However, these models did not take the influence of mechanical factors into account. Geris *et al*. considered the influences of both mechanical factors and biochemical factors^26^. However, in their work, the geometry of the callus model was simplified to a rectangular region, which cannot reflect the real boundary conditions. In addition, in this study, the rate of angiogenesis was constant, which was also not in accordance with real angiogenesis and this also needs improvement in the future.
2. Finite element analysis is one of the most popular methods for predicting the biomechanical properties of biological tissues. With the finite element method, a more physically-realistic and accurate solution can be provided by using knowledge about the soft tissue or organ (e.g. organ geometry, elastic constants and boundary conditions of the problems). Finite element analysis accuracy and calculation time are proportional to the number of nodes. The larger the number of nodes, the more accurate the solution and longer the calculation time are. However, as a prediction model that will offer help to doctors, we need the computational model to have both relatively high accuracy and quick simulation time such that doctors can make a quick decision and treat the patients as soon as possible. Therefore, once coming to the clinical application, it is limited for the use of finite element analysis. A possible alternative to the finite element method for simulating the biomechanical properties for biological tissues is machine learning. Through machine learning, a mapping function combining the input variables (e.g. external load applied to tissue, biomechanical parameters or elastic constants, and the corresponding geometry of the soft tissue) and output variables (e.g. deformation and local strain) can be estimated. Once the training process is performed, it will save more time than finite element analysis.
3. The fracture healing process is a complex biological process that is regulated by both mechanical factors and biochemical factors. In this study, we only consider the regulatory role of mechanical stimuli on fracture healing. Biochemical factors, such as growth factors, also have an important influence on the healing process. Some growth factors, such as transforming growth factor-*β* (TGF-*β*), platelet-derived growth factors (PDGFs), bone morphogenetic proteins (BMPs) and vascular endothelial growth factors (VEGF), have been demonstrated to regulate the production of chondrocytes, osteoblasts and endothelial cells^33–41^. Therefore, it will be an improvement for the current model to take the influence of biomechanical factors into account.

In all of the above, the new set-up dynamic fracture healing model allows us to simulate the fracture healing process under different IFM conditions resulting in the different mechanical conditions. This model enables us to communicate with medical experts or orthopedists easily with the fuzzy logic rules and offer help for them to optimize the treatment methods and reduce the need for animal experiments. In addition, the model has the potential to extend to a multiscale model, which can simulate the healing process in a more mechanistic way and help us understand the complex dynamic interactions between tissue differentiation with the call region.

## Methods

**Figure 6:**
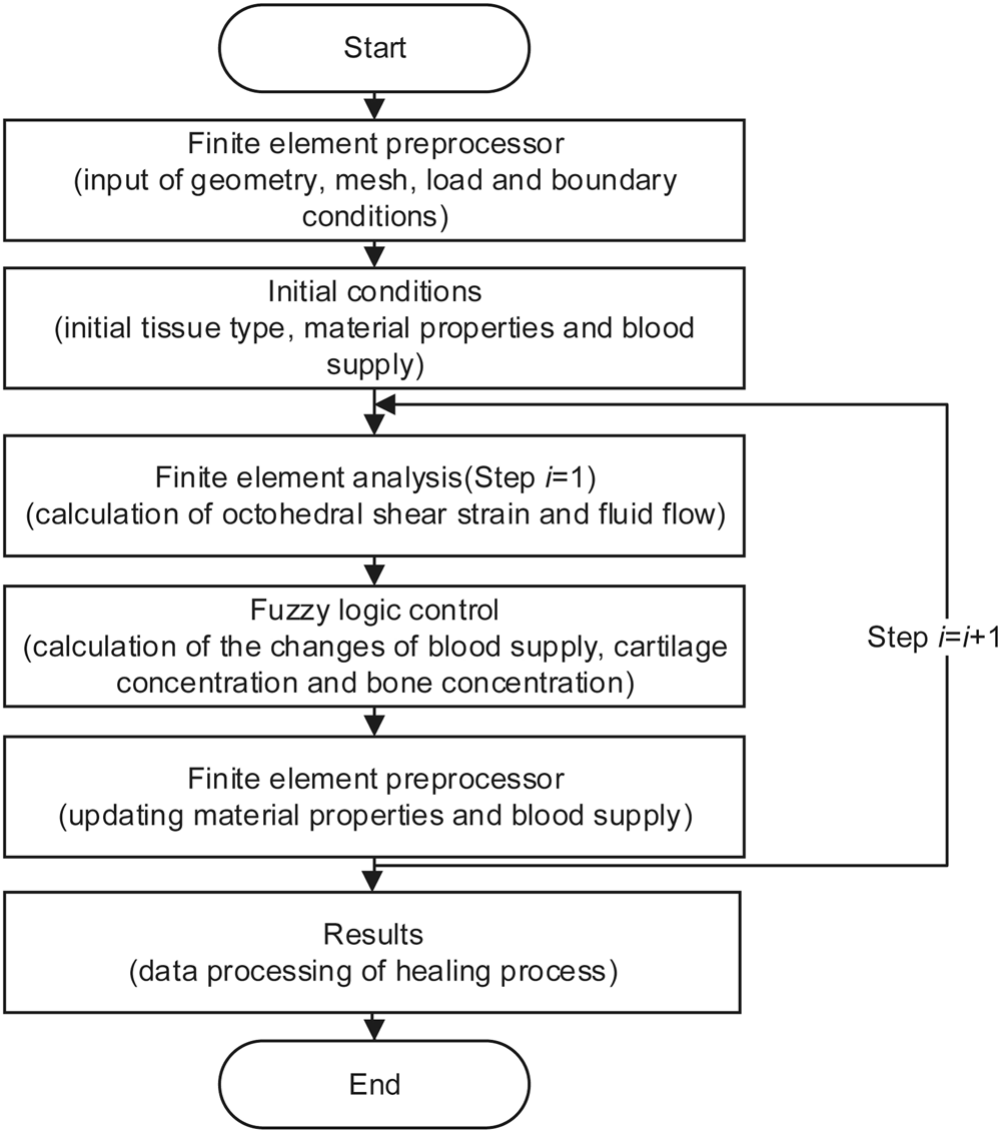
Flow chart of simulation of bone fracture healing.

The simulation of bone fracture healing can be described as an iterative process that includes biphasic poroelastic finite element analysis and fuzzy logic control, as shown in Fig. 6. The following is a detailed description of biphasic finite element analysis and fuzzy logic control, respectively.

### Calculation of biophysical stimulus

A local biophysical stimulus *S* was used to regulate the tissue differentiation in our work. Following the work of Predergast *et al*.^42^, the biophysical stimulus was defined as

1 $$S=\frac{\gamma }{a}+\frac{v}{b}$$

where *γ* is the octahedral shear strain and *v* is the fluid velocity; and *a* and *b* are empirical constants^20^. Their values are adopted from the work of Lacroix and Prendergast^11^: *a* = 3.75% and *b* = 3 *μms*^−1^.

**Figure 7:**
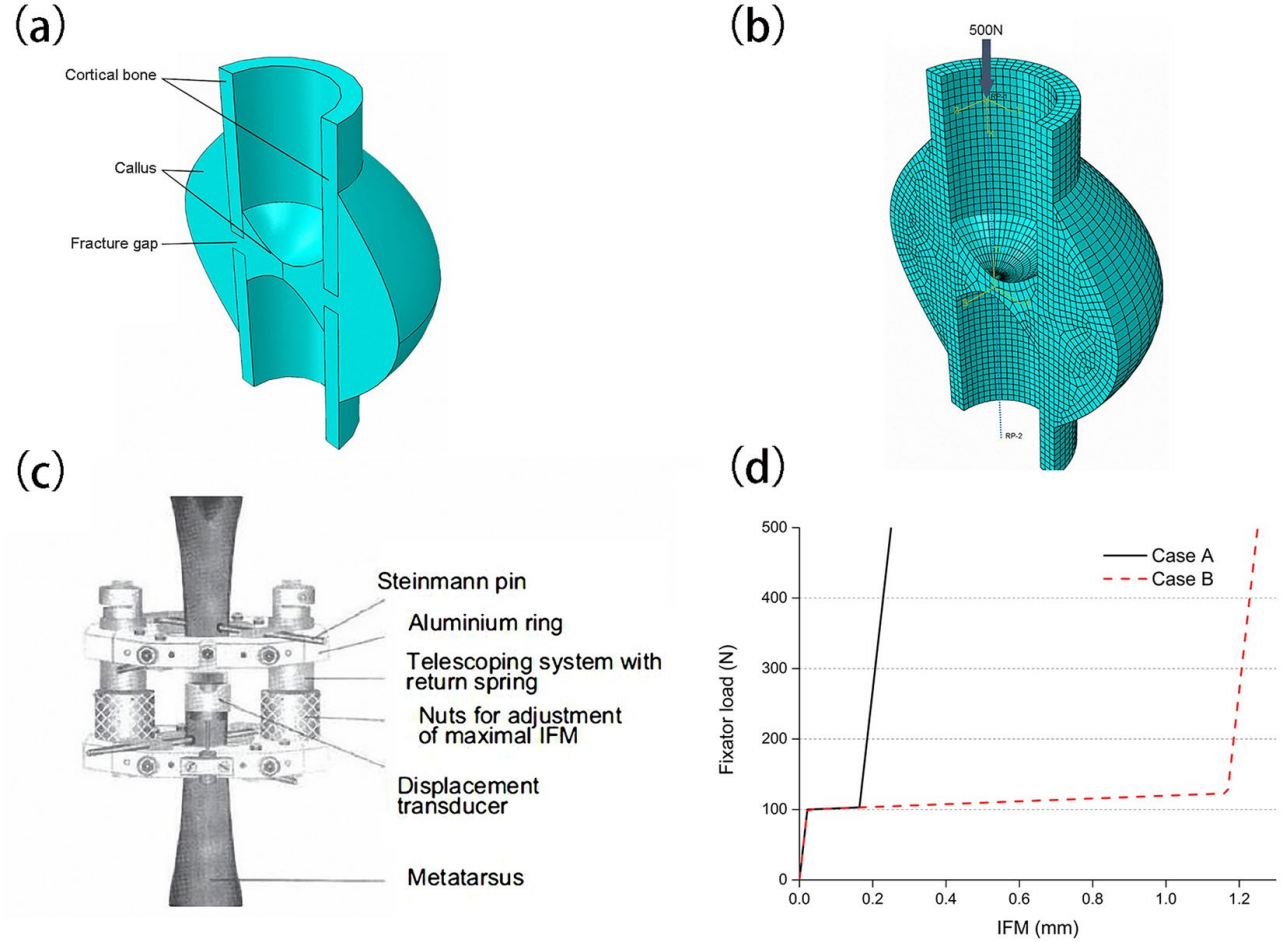
(**a**) Geometry model of bone fracture healing. (**b**) Finite element model of bone fracture healing. (**c**) Schematic drawing of a fractured bone with a custom-designed external fixator^29^. With permission from John Wiley and Sons. (**d**) The nonlinear constitutive behavior of fixator under case A and B, respectively.

For the calculation of Octahedral shear strain *γ* and fluid velocity *v*, a biphasic poroelastic finite element method was used. The method was implemented in ABAQUS 6.13–1 (Dassault Syste’mes Simulia Corp., Providence, RI, USA). A three-dimensional finite element model of an ovine tibia was modelled based on the animal models in the work of Claes *et al*.^29^. The geometric model is shown in Fig. 7a including the fracture gap, cortical bone and callus around the cortical bone. Due to the symmetrical characteristic of the callus with transverse osteotomy, half of the geometry of the callus was modelled. The finite element model (Fig. 7b) consisted of hexahedral elements and wedge elements (Case A: 13,688 elements and Case B: 15,225 elements). The callus region was filled with connective tissue at the initial stage.

A custom-designed external fixator was used to stabilize the bone fractures (Fig. 7c). The detailed information about the fixator is in the work of Claes *et al*.^43^. The nonlinear constitutive behaviour of the fixator is shown in Fig. 7d, which was adopted from the work of Simon *et al*.^7^. Due to the high bending and torsional stiffness of the fixator, the fracture ends can only move axially. Therefore, we used a translational connector in the interaction module of the ABAQUS software to model the constitutive behaviour of the fixator. First, two reference points (RP-1 and RP-2) were modelled in the top and bottom centre of cortical bone respectively. Then a point-to-point wire was established between these two points, which represents the translational connector. Finally, the nonlinear constitutive behaviour of the fixator was assigned to the wire and the modelling of the fixator was completed. A 500 N external load was applied on the RP-1 and the full restraint was applied on the RP-2. Through the finite element analysis, octahedral shear strain *γ* and fluid velocity *v* were calculated.

All finite elements had poroelastic material properties and the material properties of cortical bone, connective tissue, cartilage tissue and woven bone are shown in Table 3. As the healing process proceeded, tissue differentiation occurred within the callus region. Therefore, the callus elements were updated at each time step based on the properties of pure tissue and their current concentrations in the same element. For the element’s Young’s modulus *E*_*element*_, we used a cubic rule of mixture that is based on the experimental relationship from Carter and Hyes^44^:

2 $${E}_{element}={E}_{conn}{c}_{elem,conn}^{3}+{E}_{cart}{c}_{elem,cart}^{3}+{E}_{woven}{c}_{elem,woven}^{3}$$

where *E*_*element*_ represents element Young’s modulus; *E*_*conn*_represents Young’s modulus of connective tissue, *c*_*elem,conn*_ represents the concentration of connective tissue in an element; ${E}_{cart}$ represents Young’s modulus of cartilage, *c*_*elem,cart*_ represents the concentration of cartilage in an element; *E*_*woven*_ represents Young’s modulus of woven bone, *c*_*elem,woven*_ represents the concentration of woven bone in an element.

**Table 3:** Material properties of connective tissue, cartilage, woven bone and cortical bone.

Tissue	Young’s modulus (Mpa)	Possion’s ratio	Permeability (m^4^/Ns)	Porosity
Connective tissue	3	0.3	1E-14	0.8
Cartilage	200	0.45	5E-15	0.8
Woven bone	4000	0.36	3.7E-13	0.8
Cortical bone	10000	0.36	1E-17	0.04

For the element’s Possion’s ratio *v*_*element*_, permeability *perm*_*element*_ and porosity *poro*_*element*_, we used a linear rule of mixture:

3 $${\nu }_{element}={\nu }_{conn}{c}_{elem,conn}+{\nu }_{cart}{c}_{elem,cart}+{\nu }_{woven}{c}_{elem,woven}$$

where *v*_*element*_ represents element’s Possion’s ratio; *v*_*con*_ represents Possion’s ratio of connective tissue, *c*_*elam,conn*_ represents the concentration of connective tissue in an element; *v*_*cart*_ represents Possion’s ratio of cartilage, *c*_*elam,cart*_ represents the concentration of cartilage in an element; *v*_*women*_ represents Possion’s ratio of woven bone, *c*_*elam,woven*_ represents the concentration of woven bone in an element.

4 $$per{m}_{element}=per{m}_{conn}{c}_{elem,conn}+per{m}_{cart}{c}_{elem,cart}+per{m}_{woven}{c}_{elem,woven}$$

where *perm*_*element*_ represents element permeability; *perm*_*corn*_ represents permeability of connective tissue, *c*_*elem,conn*_ represents the concentration of connective tissue in an element; *perm*_*cart*_ represents permeability of cartilage, *c*_*elem,cart*_ represents the concentration of cartilage in an element; *perm*_*woven*_ represents permeability of woven bone, *c*_*elem,woven*_ represents the concentration of woven bone in an element.

5 $$por{o}_{element}=por{o}_{conn}{c}_{elem,conn}+por{o}_{cart}{c}_{elem,cart}+por{o}_{woven}{c}_{elem,woven}$$

where *poro*_*element*_ represents element porosity; *poro*_*conn*_ represents porosity of connective tissue, *c*_*elem,conn*_ represents the concentration of connective tissue in an element; *poro*_*cart*_ represents porosity of cartilage, *c*_*elem,cart*_ represents the concentration of cartilage in an element; *poro*_*woven*_ represents porosity of woven bone, *c*_*elem,woven*_ represents the concentration of woven bone in an element.

Connective tissue concentration, cartilage concentration and bone concentration in the callus region has the following relationship:

6 $${c}_{conn}+{c}_{cart}+{c}_{woven}\mathrm{=1}$$

where *c*_*onn*_ represents connective tissue concentration in an element; *c*_*cart*_ represents cartilage concentration in an element; *c*_*woven*_ represents woven bone concentration in an element.

### Simulation of tissue differentiation

**Figure 8:**
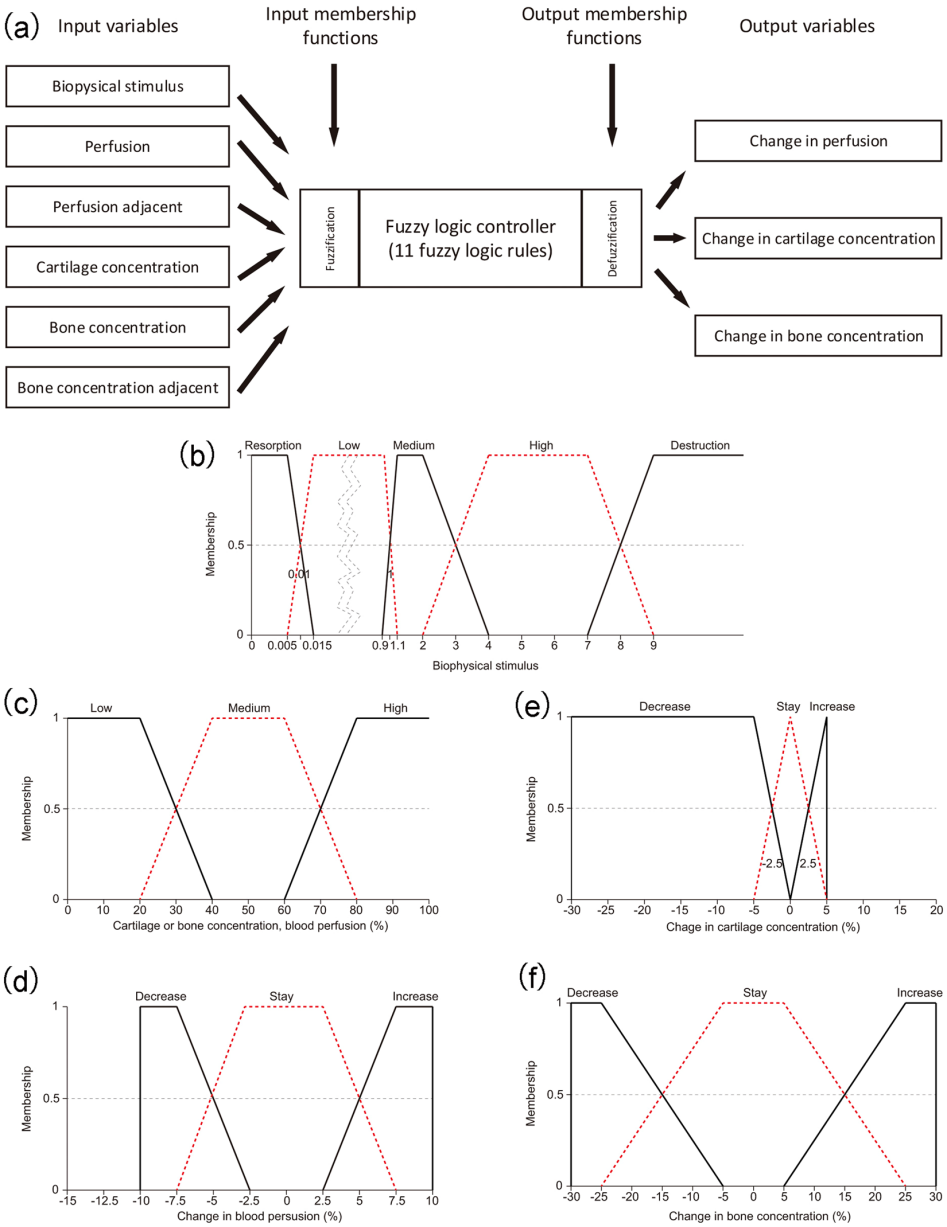
(**a**) Fuzzy logic controller of tissue differentiation within the callus region. The controller has six input variables and three output variables. The fuzzification module transforms numerical values to associated linguistic values and the defuzzigication module transforms linguistic values to associated numerical values. (**b**) Membership function of biophysical stimulus. (**c**) Membership functions of perfusion, perfusion in an adjacent element, cartilage concentration, bone concentration and bone concentration in an adjacent element. (**d**) Membership function of change in perfusion. (**e**) Membership function of change in cartilage concentration. (**f**) Membership function of change in bone concentration. Among these membership functions, (**b**) and (**c**) represent the membership function of input variables. (**d**), (**e**) and (**f**) represent the membership function of output variables.

Fuzzy logic control was used to simulate the process of tissue differentiation inside the fracture callus. In our work, the Fuzzy Inference Engine that is provided by the Fuzzy Toolbox in MATLAB R2011A (The MathWorks, Inc., Natick, MA, USA) was employed to simulate biological processes. The Mamdani-type fuzzy logic controller was used to predict the changes of blood perfusion, cartilage concentration and bone concentration. The controller consisted of six input variables and three output variables (Fig. 8a). The input variables included biophysical stimulus, perfusion, perfusion in an adjacent element, cartilage concentration, bone concentration and bone concentration in an adjacent element. The output variables included change of perfusion, change of cartilage concentration and change of bone concentration.

The fuzzy controller consisted of 14 linguistic if-then rules that described the processes of angiogenesis, intramembranous ossification, chondrogenesis, endochodral ossification and tissue destruction (Table 4). Rules no. 1–3 described the process of angiogenesis, which depended on the local biophysical stimulus and perfusion conditions of adjacent elements. The moderate biophysical stimulus and higher perfusion in at least one adjacent element increased the perfusion of the current element. Rule no. 4 described the process of intramembranous ossification. The bone concentration increased when the biophysical stimulus was low, perfusion was high, cartilage was low and bone concentration was high in at least one adjacent element. Rules no. 5–8 described the process of chondrogenesis, which depends on the local biophysical stimulus and current cartilage concentration. Rules no. 9–12 described the process of endochodral ossification. When the local biophysical stimulus was appropriate and the perfusion was sufficient, this process was active, which led to the increase of bone concentration and decrease of cartilage concentration. Rule no.13 modelled the overloading conditions during fracture healing. When active, there was a decrease in perfusion, cartilage concentration and bone concentration. To make the fuzzy logic rules work, rule no. 14 was added.

**Table 4:** Fuzzy logic rules for tissue differentiation within callus region.

Rules	Input variables						Output variables		
	*S*	*c*_*perfusion*_	*c*_*andPerfusion*_	*c*_*cartilage*_	*c*_*bone*_	*c*_*adbone*_	Δ*c*_*perfusion*_	Δ*c*_*cartilage*_	Δ*c*_*bone*_
1	Not destruction	Low	Not low	—	—	—	Increase	—	—
2	Not destruction	Medium	High	—	—	—	Increase	—	—
3	Not destruction	High	—	—	—	—	Increase	—	—
4	Low	High	—	Low	—	High	—	—	Increase
5	High	—	—	Low	Not high	—	—	Increase	—
6	Low	—	—	Low	Not high	—	—	Increase	—
7	Medium	—	—	Not low	—	—	—	Increase	—
8	Low	—	—	Not low	—	—	—	Increase	—
9	Low	Not low	—	Not low	—	Not low	—	Decrease	Increase
10	Medium	Not low	—	Not low	—	Not low	—	Decrease	Increase
11	Low	Not low	—	Not low	—	High	—	Decrease	Increase
12	Low	Not low	—	Low	High	High	—	Decrease	Increase
13	Destruction	—	—	—	—	—	Decrease	Decrease	Decrease
14	Not destruction	—	—	—	—	—	Stay	Stay	Stay

Note: “—” represent the current variable doesn’t participate in this rule. *S* represents the biophysical stimulus of current element. *c*_*perfusion*_ represents the perfusion concentration of current element. *c*_*adPerfusion*_ represents the highest perfusion concentration of adjacent elements of current element. *c*_*cartilage*_ represents the cartilage concentration of current element. *c*_*bone*_ represents the bone concentration of current element. *c*_*adbone*_ represents the highest bone concentration of adjacent elements of current element. Δ*c*_*perfusion*_ represents the change of perfusion concentration of current element. Δ*c*_*cartilage*_ represents the change of cartilage concentration of current element. Δ*c*_*bone*_ represents the change of bone concentration of current element.

Membership functions of the six input variables and three output variables were defined as trapezoidal functions (Fig. 8b–f). Through the membership functions, the quantitative values (biophysical stimulus, tissue concentrations and perfusion) were switched to linguistic values (e.g. low, medium, high, increase and decrease). The centroid method was used for the fuzzy inference procedure, which calculated the final output prediction as an average sum of the weighted single outputs of the active rules. The results of cell culture experiments from the work of Kaspar *et al*.^45^ served as the basis for defining the membership functions of tissue concentrations and perfusion (Fig. 8c–f). Following the work of Lacroix and Prendergast11, the membership function of biophysical stimulus was defined (Fig. 8b).

The initial conditions for perfusion were defined according to the work of Simon *et al*.^7^. At the initial stage, the perfusion in the callus region was set to 0%. At the peripheral boundary of the callus, the perfusion was set to 30%, which represented blood supply from adjacent soft tissues^46^. After ten days, the perfusion in the medullary channel was set to 30%, which represented a revascularisation from the marrow^46^.

### Mesh convergence study

**Figure 9:**
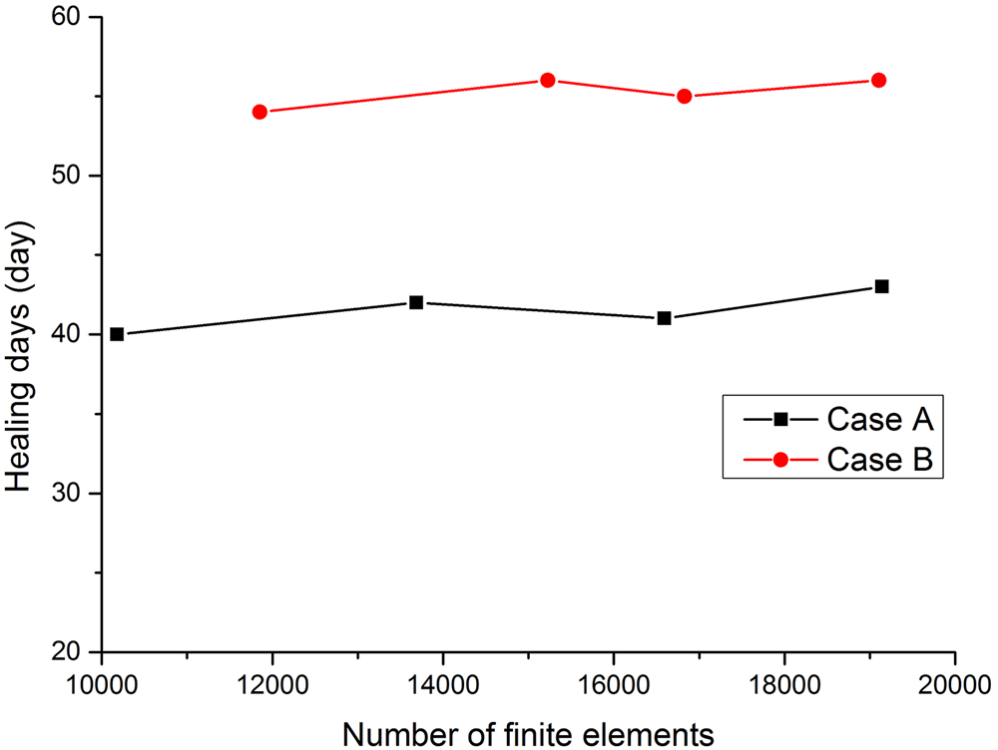
Mesh convergence analysis for case A and case B. Influence of number of finite elements on predicted healing days for both cases A and B.

To test whether the mesh size affect the results of fracture healing simulation results (healing days), we conducted a mesh convergence study, which is shown in Fig. 9. From the Fig. 9, we can concluded that with the increase of element numbers, there is a small influence on the healing days, which is acceptable. Therefore, for the computation model, we thick that the element size has no relationship with healing simulation.

### Data availability

The datasets generated during and/or analysed during the current study are available from the corresponding author on reasonable request.
